# Enhanced inflammatory responses to toll-like receptor 2/4 stimulation in type 1 diabetic coronary artery endothelial cells: the effect of insulin

**DOI:** 10.1186/1475-2840-9-90

**Published:** 2010-12-16

**Authors:** Jilin Li, Chunhua Jin, Joseph C Cleveland, Lihua Ao, Dingli Xu, David A Fullerton, Xianzhong Meng

**Affiliations:** 1 Department of Surgery, University of Colorado Denver, Aurora, Colorado, USA; 2 Department of Cardiology and the Core Laboratory for Organ Failure Research, Nanfang Hospital, Southern Medical University, Guangzhou, China; 3 Center for Laboratory Medicine, Southern Medical University, Guangzhou, China

## Abstract

**Background:**

Endothelial inflammatory responses mediated by Toll-like receptors (TLRs), particularly TLR2 and TLR4, play an important role in atherogenesis. While Type 1 diabetes (T1D) promotes the development and progression of atherosclerosis, the effect of T1D on TLR2/4-mediated inflammatory responses in coronary artery endothelial cells (CAECs) remains unclear.

**Methods:**

We tested the hypothesis that diabetic CAECs have enhanced inflammatory responses to TLR2/4 stimulation. Non-diabetic and diabetic CAECs were treated with TLR2 agonist peptidoglycan and TLR4 agonist lipopolysaccharide. The expression of ICAM-1, IL-6 and IL-8 were analyzed by real-time PCR, immunoblotting and ELISA, and NF-κB activation by immunoblotting and immunostaining. In additional experiments, insulin was added before TLR stimulation to determine whether insulin deficiency alone is responsible for the alteration of TLR2/4-mediated inflammatory responses.

**Results:**

Stimulation of TLR2 or TLR4 induced NF-κB activation, and the expression of ICAM-1, IL-6 and IL-8. Interestingly, the expression of inflammatory mediators was significantly enhanced in diabetic cells. The enhanced inflammatory responses correlated with augmented NF-κB activation in the absence of a change in TLR2 or TLR4 protein levels. Further, pretreatment of diabetic cells with insulin failed to suppress the enhanced inflammatory responses.

**Conclusions:**

Diabetic CAECs have enhanced inflammatory responses to stimulation of TLR2 or TLR4, and insulin alone is insufficient to correct the hyper-inflammatory responses. The mechanism underlying the enhanced inflammatory responses appears to be augmentation of pro-inflammatory signaling, rather than up-regulation of levels of TLR2 and TLR4. These findings suggest that diabetic CAECs adopt a hyper-inflammatory phenotype and that this endothelial phenotypic change may predispose coronary artery to atherogenesis.

## Background

Growing evidence supports an important role for vascular local inflammation in the development and progress of atherosclerosis [1-3]. Endothelial cells are important in immune and inflammatory responses [4,5], and inflammatory activation of the endothelial cells is a critical step in the development of atherosclerosis [1,3]. In the Type 1 diabetes (T1D) population, atherogenesis occurs in younger ages and advances faster [6,7]. However the underlying mechanisms are incompletely understood.

Toll-like receptors (TLRs) are pathogen pattern recognition receptors that recognize bacterial and viral products, and other pathogens [8]. Activation TLR2 or TLR4 by microbial ligands induce a cascade of intracellular signaling events, culminating in the production of pro-inflammatory mediators. Thus, TLR2 and TLR4 have a central role in innate immunity and inflammation [9]. A number of studies demonstrate that these two major innate immune receptors play a mechanistic role in the development of atherosclerosis [10-12]. In addition, TLR2 ligand peptidoglycan (PGN) and TLR4 ligand lipopolysaccharide (LPS) have been found in vessels with early atherosclerotic lesions [13,14]. While these bacterial agents induce the production of multiple pro-inflammatory mediators in mononuclear cells [15,16], their effects on the inflammatory responses in coronary artery endothelial cells (CAECs) remain to be determined. Investigation of the effect of T1D on CAEC inflammatory responses to TLR2/4 stimulation could provide insights into the mechanisms underlying the pro-atherogenic phenotype associated with this disease.

TLR2 and TLR4 have also been implicated in the pathophysiology of T1D. In an experimental T1D model, TLR2 is involved in the autoimmune inflammation in the pancreatic islet [17]. The expression of TLR2, as well as TLR3, TLR4 and TLR5 in bone marrow-derived macrophages is increased in diabetic NOD mice [18]. Insulin is found to suppress the expression of TLR2 in mononuclear cells at the transcriptional level [19]. In addition, altered TLR4 function is involved in the inflammation in B cells from diabetes mellitus patients by two mechanisms: elevation of pro-inflammatory IL-8 and lack of anti-inflammatory/protective IL-10 production [20]. While these studies indicate altered cellular TLR expression and responses associated with T1D, it remains unclear whether TLR2/4 levels and the inflammatory responses to TLR2/4 agonists are altered in CAECs from T1D patients.

We hypothesized that CAECs of T1D patients have enhanced inflammatory responses to TLR2/4 stimulation. The purposes of this study are to determine: 1) the effect of PGN and LPS on the inflammatory responses in human CAECs, 2) whether TLR2/4 levels, signaling and TLR2/4-mediated expression of pro-inflammatory mediators are altered in CAECs from T1D patients, and 3) the effect of insulin on the inflammatory responses in diabetic CAECs.

## Methods

### Chemicals and reagents

Staphylococcus aureus PGN and E coli LPS were purchased from Sigma-Fluka (St. Louis, MO, USA). Human CAECs from non-diabetic and diabetic donors (information in Table 1) were purchased from Lonza (Boulder, CO, USA). Protein assay reagents and ECL immunoblotting substrate were purchased from Pierce (Rockford, IL, USA). The following antibodies were used for Western-blot analysis: rabbit anti-human intercellular adhesion molecule (ICAM)-1 (Santa Cruz Biotechnology, Santa Cruz, CA, USA), monoclonal rabbit anti-human TLR2 (Imgenex, San Diego, CA, USA), monoclonal rabbit anti-human TLR4 (Santa Cruz Biotechnology, Santa Cruz, CA, USA), rabbit anti-human phosphor-nuclear factor kappa B (NF-κB) p65 (Cell Signaling, Boston, MA), rabbit anti-human total NF-κB p65 (Cell Signaling, Boston, MA, USA), rabbit anti-human beta-actin (Cell Signaling, Boston, MA, USA), and rabbit anti-mouse ICAM-1 (Santa Cruz Biotechnology, Santa Cruz, CA, USA). RNeasy micro kit was purchased from QiaGen (Valencia, CA, USA). IL-6 and IL-8 ELISA kits were purchased from R&D Systems (Minneapolis, MN, USA).

**Table 1:** Details of the human CAECs used in this study

*Cells used*	*Lot number*	*Donor*	*Initiation passage*	*Passage used*
Non-diabetic	3F0239	25y, ♂	3	4-6
Non-diabetic	7F4019	56y, ♂	3	4-6
Non-diabetic	7F4249	36y, ♂	3	4-6
T1D	7F3422	56y, ♂	3	4-6
T1D	7F4021	57y, ♂	3	4-6
T1D	7F3795	34y, ♂	3	4-6

### Animals

TLR2 knockout (TLR2 KO), C57BL/6 (WT), and C3H/HeJ (TLR4-defective) mice were purchased from Jackson Laboratory (Bar Harbor, MA, USA), and male C3H/HeN (TLR4-competent) mice were purchased from Charles River Laboratories (Wilmington, MA, USA). The mice were 12 weeks old and acclimated in a quarantine room for 2 weeks before experiments, and maintained on a standard pellet diet. Their body weight was 23 to 28 g when used for the experiments. All experiments were approved by the Animal Care and Research Committee of the University of Colorado Denver, and this investigation conforms to *The Guide for the Care and Use of Laboratory Animals *(National Research Council, revised 1996).

### Culture of human CAECs

Cells were grown in endothelial cell growth medium (EBM-2 from Lonza, Boulder, CO, USA) supplemented with EGM-2 (2% fetal cattle serum, hydrocortisone, human fibroblast growth factor, vascular endothelial growth factor, insulin-like growth-factor, ascorbic acid, epidermal growth factor, gentamicin/amphotericin-1000, and heparin).

For the experiments, cells were seeded in 500 μl complete medium in 24-well plates. After growing to confluence, medium was changed completely. PGN and LPS was diluted in complete cell culture medium and added to the cells. The final concentrations of PGN and LPS were 10 μg/ml and 200 ng/ml, respectively. In additional experiments, human insulin (from Sanofi Aventis, Bridgewater, NJ, USA, final concentration 10 or 100 U/l) was added to the cells 1 h prior to adding PGN or LPS.

### Isolation and culture of mouse coronary vascular endothelial cells

Mouse coronary vascular endothelial cells were isolated according to Li's method [21]. Briefly, hearts were briefly dipped into 70% ethanol to devitalize epicardial mesothelial cells and endocardial endothelial cells. Ventricular tissue was minced into approximately 1.0 mm^3 ^pieces, and digested at 37°C for 10 min in 2.0 ml of norminally calcium-free Hank's balanced salt solution (HBSS) supplemented with collagenase II (1.0 g/l, glucose (2.0 g/l), taurine (2.5 g/l), bovine serum albumin (BSA, 0.1%), and MgCl_2 _(1.4 mM). Then, the tissue pellet was re-suspended in a second digestion solution containing 0.125% trypsin, 0.1 mM EDTA and 2.0 g/l glucose dissolved in HBSS, and incubated at 37°C for 10 min with shearing by pipetting once every 3 min. At the end of this digestion, the supernatant was transferred into a 15 ml Falcon tube containing 1.0 ml of FBS, and cells were separated from tissue debris and remaining myocytes by spinning at 500 rpm for 5 min. The supernatant was centrifuged at 1,200 rpm (4°C) for 8 min to collect endothelial cells. The cells were resuspended in 10 ml Dulbecco's modified Eagle's medium (DMEM) supplemented with 20% FCS, penicillin (50,000U/l) and streptomycin (50 g/l). Cells were seeded in 500 μl complete medium in 24-well plates and cultured at 37°C for 2 h. Then, non-attached cells were removed by changing medium. Cells of 90% confluence were treated with PGN or LPS as described for human cells.

### Immunoblotting

Immunoblotting was used to detect ICAM-1, phosphorylated NF-κB p65, total NF-κB p65, TLR2, TLR4 and beta-actin. After treatment, CAECs were washed three times with cold PBS, and then lysed with lysis buffer (protease inhibitor cocktail and Mammalian Protein Extraction Reagent, Thermo Scientific, Waltham, MA, USA). Samples were separated on 4-20% SDS-polyacrylamide gels (Bio-Rad, Hercules, CA, USA) and transferred onto nitrocellulose membranes. Membranes were blocked for 1 h at room temperature with 5% dry milk in TPBS (PBS containing 0.1% Tween 20), and then incubated with the appropriate primary antibodies (ICAM-1 antibody was diluted 1:200, beta-actin 1:1000, and all others 1:500) overnight at 4°C. After washing with TPBS, membranes were incubated with horseradish peroxidase (HRP)-linked secondary antibodies (1:5000 dilution with TPBS containing 5% dry milk) at room temperature for 1 h. Bands were developed using ECL and exposed on X-ray films. Band density was analyzed using NIH ImageJ software.

### Cytokine ELISA

Cytokine concentrations in cell culture supernatants were quantified by ELISA kits (R&D Systems, Minneapolis, MN, USA) as previously reported [22]. Recombinant cytokines were used to construct standard curves. Absorbance of standards and samples was determined spectrophotometrically at 450 nm using a microplate reader (Bio-Rad, Hercules, CA, USA). Results were plotted against the standard curve. The assays were carried out according to the protocols provided by the manufacturer.

### Immunofluorescent staining

Immunofluorescent staining was performed as previously reported to examine NF-κB intranuclear translocation in CAECs [23]. Briefly, cells were cultured in 8-well chamber slides to 40-50% confluence. Cells were stimulated with PGN or LPS for 30 or 60 min. After washing with cold PBS, cells were treated with a mixture of 30% methanol and 70% acetone at room temperature for 5 min and fixed in PBS-buffered 3.5% paraformaldehyde at room temperature for 10 min. After washing with PBS, cells were blocked with 10% donkey serum for 30 min. Then, cells were incubated for 2 h with a rabbit polyclonal antibody against NF-κB p65. Control cells were incubated with non-immune rabbit IgG. After washing with PBS, cells were incubated Cy3-conjugated donkey anti-rabbit IgG for 1 h to label NF-κB p65 red. Nuclei were counter-stained blue with bis-benzimide. Photography was performed with a Leica DMRX microscope (Wetzlar, Germany).

### RNA-isolation and real-time RT-PCR

Confluent CAECs were treated with PGN or LPS for 1 or 2 h. Thereafter cells were harvested with lysis buffer, and mRNA was extracted using a Qiagen RNeasy Mini Kit (Valencia, CA, USA). cDNA was prepared by reverse transcription (SuperScript III First-Strand, Invitrogen, Carlsbad, CA, USA). Real-time PCR was performed as previously reported using Power Sybr Green PCR Master Mix (Applied Biosystems, Foster City, CA, USA) with Corbbet Cycler (Qiagen, Valencia, CA, USA) [24]. Each reaction was carried out for 45 or 50 cycles in a total volume of 15 μl (4.9 μl H2O, 7.5 μl Sybr Green Mix, 0.3 μl of each 5 mM primer, 2.0 μl cDNA). The following sets of primers were used to amplify specific cDNA fragments: GAPDH (forward: 5'-GGC TCT CCA GAA CAT CAT CC; reverse:5'-TTT CTA GAC GGC AGG TCA GG-3'); ICAM-1 (forward: 5'-AGC TTC TCC TGC TCT GCA AC; reverse: 5'-GTC TGC TGG GAA TTT TCT GG); IL-8 (forward:5'-CTC TTG GCA GCC TTC CTG ATT; reverse: 5'-TAT GCA CTG ACA TCT AAG TTC TTT AGC A); IL-6 (forward: 5'-CAT CCA TCT TTT TCA GCC ATC TTT; reverse: TGA CAA ACA AAT TCG GTA CAT CCT).

### Statistics

Data are expressed as mean ± standard error of mean (SEM). Analysis of variance (ANOVA) was performed, and differences were considered significant when *P *< 0.05, as verified by Fisher post-hoc test.

## Results

### Diabetic CAECs express higher levels of ICAM-1 in response to stimulation of TLR2 and TLR4

**Figure 1:**
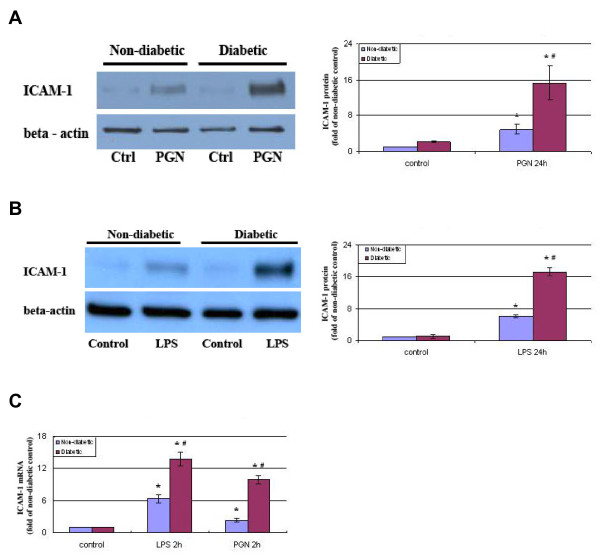
**Diabetic CAECs express higher levels of ICAM-1 after stimulation with PGN and LPS**. **A and B**. Non-diabetic and diabetic CAECs were stimulated with PGN (10 μg/ml) or LPS (200 ng/ml) for 24 h, and ICAM-1 protein levels were analyzed by immunoblotting. Diabetic cells exhibited a greater increase in ICAM-1 protein levels. **C**. Non-diabetic and diabetic CAECs were stimulated with PGN (10 μg/ml) or LPS (200 ng/ml) for 2 h and analyzed for ICAM-1 mRNA levels by real time RT-PCR. Diabetic cells expressed higher levels of ICAM-1 mRNA. Results are expressed as Mean ± SEM; n = 5; *P < 0.05 vs. control; #*P *< 0.05 vs. non-diabetic cells treated with LPS or PGN.

We determined the effects of PGN and LPS on ICAM-1 expression in non-diabetic and T1D CAECs. Stimulation of cells with PGN or LPS induced the expression of ICAM-1 in both non-diabetic and diabetic CAECs (Figure 1A and 1B). While ICAM-1 protein levels increased by 4.9 folds in non-diabetic cells, it increased by 6.9 folds in diabetic cells following PGN stimulation (Figure 1A). Similarly, LPS stimulation resulted in a more robust increase in ICAM-1 protein levels in diabetic cells (Figure 1B). Further, diabetic cells exhibited a greater increase in ICAM-1 mRNA levels after stimulation with either PGN or LPS (Figure 1C). Therefore, diabetic CAECs have enhanced ICAM-1 responses to PGN and LPS.

**Figure 2:**
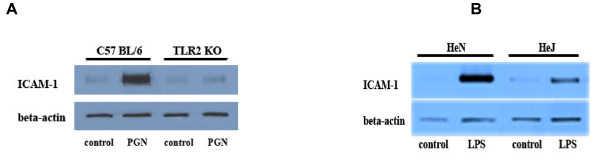
**Induction of ICAM-1 expression in coronary endothelial cells by PGN and LPS requires TLR2 and TLR4, respectively**. **A**. Coronary endothelial cells isolated from TLR2 KO and wild type (C57BL/6) mice were treated with PGN (10 μg/ml) for 24 h. A representative immunoblot shows that PGN induced a robust increase in ICAM-1 levels in wild type cells, but it had a minimal effect on ICAM-1 levels in TLR2 KO cells. **B**. A representative immunoblot shows that induction of ICAM-1 expression by LPS (200 ng/ml, 24 h) is markedly reduced in coronary endothelial cells from TLR4-defective (C3H/HeJ) mice.

We examined whether PGN and LPS exert an effect on coronary vascular endothelial cells through TLR2 and TLR4, respectively. We stimulated mouse coronary vascular endothelial cells with PGN or LPS for 24 h and examined cellular ICAM-1 protein levels. As shown in Figure 2, stimulation with PGN increased ICAM-1 levels by 6.3 folds in coronary vascular endothelial cells from wild-type mice, and LPS induced a 9.0-fold increase in cellular ICAM-1 levels. In contrast, the effect of PGN was essentially absent in TLR2 KO cells, and effect of LPS was markedly reduced in TLR4-defective cells. Thus, PGN induces an inflammatory response in coronary vascular endothelial cells through TLR2, and the effect of LPS is TLR4-dependent.

### Diabetic CAECs release greater amounts of IL-6 and IL-8 in response to stimulation of TLR2 or TLR4

**Figure 3:**
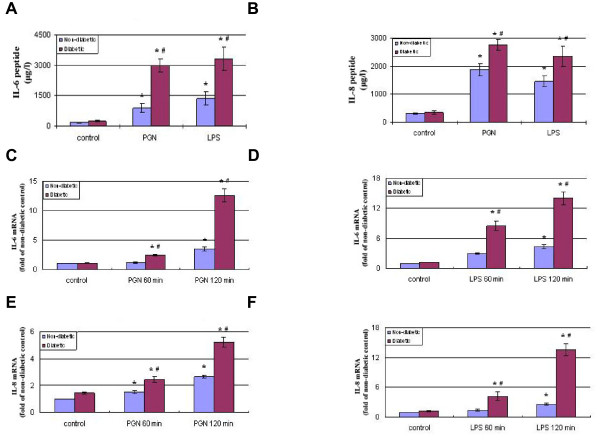
**Diabetic CAECs produce and release greater amounts of IL-6 and IL-8 in response to TLR2 and TLR4 stimulation**. Non-diabetic and diabetic CAECs were stimulated with PGN (10 μg/ml) and LPS (200 ng/ml) for 1, 2 or 24 h. Levels of IL-6 and IL-8 peptides (**A and B**) in medium were assessed by ELISA after treatment for 24 h, and mRNA levels in cell lysates (**C, D, E and F**) were analyzed with real-time PCR after treatment for 1 or 2 h. After stimulation of either TLR2 or TLR4, diabetic cells expressed higher levels of IL-6 and IL-8 mRNA and released greater amounts of IL-6 and IL-8. Results are expressed as Mean ± SEM; n = 5; *P < 0.05 vs. control; #*P *< 0.05 vs. non-diabetic cells treated with LPS or PGN.

We analyzed IL-6 and IL-8 levels in culture supernatants with or without exposing CAECs to PGN or LPS for 24 h. Interestingly, diabetic cells released more IL-6 (248.7 ± 36.9 vs. 165.1 ± 27.9 in non-diabetic cells) and IL-8 (348.2 ± 38.2 vs. 132.6 ± 10.9 in non-diabetic cells) in baseline although the differences from the baseline levels in non-diabetic cells were not significant. The release of IL-6 and IL-8 peptides increased in non-diabetic and diabetic cells following stimulation with PGN or LPS (Figure 3A). However, IL-6 and IL-8 levels in the supernatants of diabetic CAECs were 3.36 and 1.48 folds, respectively, of those of non-diabetic CAECs following stimulation of TLR2, and IL-6 and IL-8 levels following TLR4 stimulation were 1.44 and 0.63 folds higher, respectively, in diabetic cells. The enhanced release of IL-6 and IL-8 peptides in diabetic cells correlated with augmented expression of IL-6 and IL-8 mRNA at 1 and 2 h of TLR2/4 stimulation, as revealed by real-time RT-PCR (Figure 3B). Together, these results show that T1D CAECs have enhanced inflammatory responses to stimulation TLR2 and TLR4.

### TLR2 and TLR4 levels are unaltered in T1D CAECs

**Figure 4:**
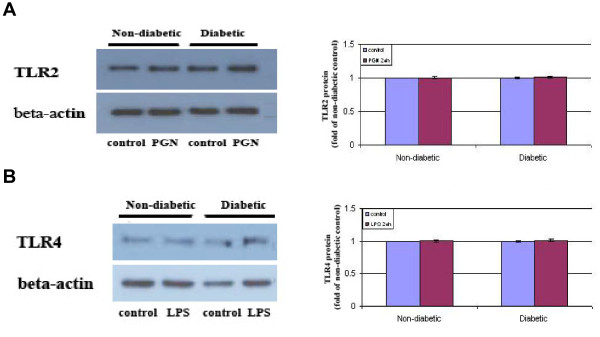
**TLR2 and TLR4 protein levels in diabetic CAECs are not altered**. Non-diabetic and diabetic CAECs were untreated or stimulated with PGN (10 μg/ml) or LPS (200 ng/ml) for 24 h. Representative immunoblots shows comparable TLR2 (**A**) and TLR4 (**B**) levels in non-diabetic and diabetic cells with and without stimulation. Results are expressed as Mean ± SEM; n = 5.

We examined whether the enhanced inflammatory responses to TLR2/4 stimulation are associated with elevated levels of these two receptors in T1D CAECs. Representative immunoblots in Figure 4 show that levels of TLR2 and TLR4 proteins were comparable between non-diabetic cells and diabetic cells in conditions with and without receptor agonists. It appears that the enhanced inflammatory responses to TLR2/4 stimulation in diabetic cells are not due to alterations in the protein levels of these two innate immune receptors.

### NF-κB activation is augmented in diabetic CAECs following stimulation of TLR2 and TLR4

**Figure 5:**
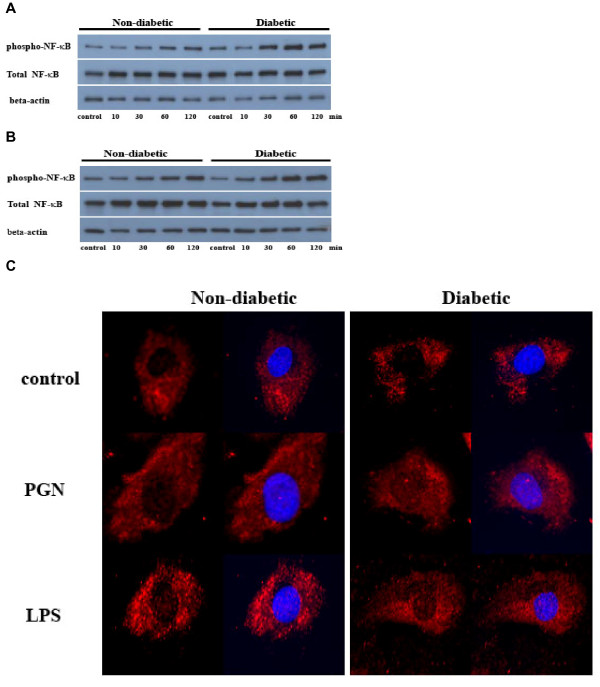
**NF-κB activation by TLR2 and TLR4 is augmented in diabetic CAECs**. Non-diabetic and diabetic CAECs were stimulated with PGN (10 μg/ml) and LPS (200 ng/ml) for 10, 30, 60 or 120 min. **A and B**. Representative immunoblots show that stimulation of either TLR2 or TLR4 induced greater phosphorylation of NF-κB p65 in diabetic cells at 30 to 120 min. **C**. NF-κB p65 was label red by immunostaining with a specific antibody, and nuclei were counter-stained blue with bis-benzimide. Representative immunofluorescent images show more intranuclear NF-κB p65 in diabetic cells at 60 min after stimulation of TLR2 or TLR4.

To understand the mechanism underlying the enhanced inflammatory responses to TLR2/4 stimulation in diabetic CAECs, we examined NF-κB phosphorylation and intranuclear translocation. Stimulation of TLR2 or TLR4 induced greater phosphorylation of NF-κB p65 in diabetic CAECs at 30 to 120 min (Figure 5A and 5B). Similarly, diabetic CAECs exhibited more pronounced intranuclear NF-κB p65 after stimulation of TLR2 and TLR4 (Figure 5C, images at 60 min are shown). The results show that the enhanced inflammatory responses to stimulation of TLR2/4 in diabetic CAECs are associated with augmented pro-inflammatory signaling.

### Insulin alone fails to suppress the inflammatory responses to TLR2 and TLR4 stimulation in T1D CAECs

**Figure 6:**
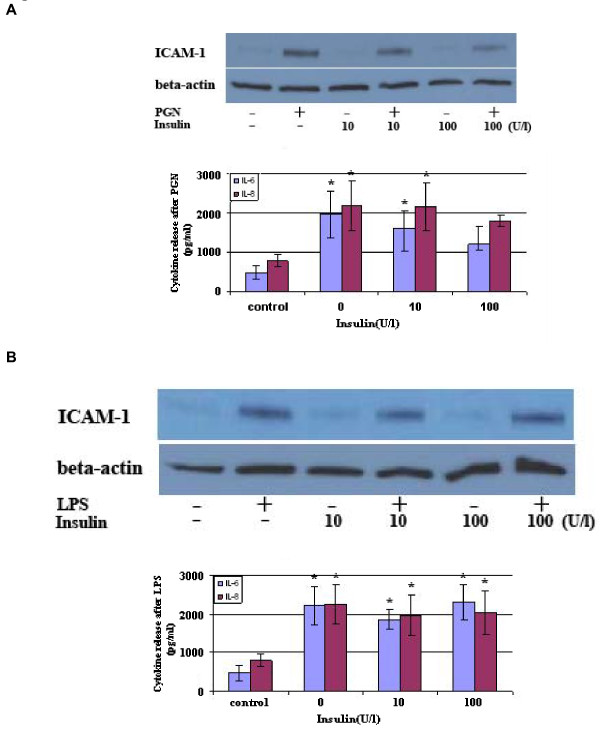
**Insulin has a minor effect on the hyper-inflammatory responses to TLR2 stimulation, but does not affect those to TLR4 stimulation in diabetic CAECs**. Insulin (10 or 100 U/l) was added to diabetic CAEC cultures 1 h prior to addition of PGN (10 μg/ml) or LPS (200 ng/ml). Cellular ICAM-1, IL-6 and IL-8 levels were analyzed at 24 h after addition of PGN or LPS. Insulin in a concentration of 10 U/l had no effect on ICAM-1, IL-6 and IL-8 levels following stimulation with either PGN or LPS. Higher concentration of insulin reduced ICAM-1 and IL-6 levels after stimulation with PGN (**A**), but did not affect LPS-induced production of ICAM-1, IL-6 and IL-8 (**B**). Results are expressed as Mean ± SEM; n = 5; *P < 0.05 vs. control.

Insulin has been found to have an anti-inflammatory effect in macrophages [25]. We determined the effect of insulin on the enhanced inflammatory response in diabetic CAECs. Human insulin was added to culture medium, in final concentrations of 10 or 100 U/l, 1 h prior to the addition of PGN or LPS. As shown in Figure 6, insulin at 10 U/l (approximately 400 folds of normal blood insulin levels) had no effect on ICAM-1, IL-6 and IL-8 levels following stimulation with either PGN or LPS. Further, insulin at 100 U/l did not affect LPS-induced production of ICAM-1, IL-6 and IL-8 although this higher concentration of insulin reduced ICAM-1 and IL-6 levels after stimulation with PGN (Figure 6). Thus, treatment with insulin alone is insufficient to correct the hyper-inflammatory phenotypic change in diabetic CAECs.

## Discussion

In this study, we demonstrated that diabetic CAECs have enhanced inflammatory responses to TLR2 and TLR4 agonists with increased expression of ICAM-1, IL-6 and IL-8. The hyper-inflammatory phenotype of diabetic CAECs is characterized by augmented NF-κB activation in response to TLR2/4 agonists in the absence of altered cellular TLR2/4 levels. Insulin alone is insufficient to correct the hyper-inflammatory responses in T1D CAECs.

### Diabetic CAECs have enhanced inflammatory responses to TLR2/4 agonists

The innate immunity is the first line of defense against microorganisms and also plays an important role in modulating the adaptive immune responses [26,27]. TLRs, acting as pathogen-recognition receptors, are important components of the innate immune system [8,28]. Endothelial innate immune responses are key events in vascular inflammation and the development of atherosclerosis [29]. Previous studies have examined the TLR4-mediated endothelial inflammatory responses [30,31]. In this study, we present novel findings that stimulation of TLR2 in human CAECs with bacterial PGN also induces the expression of adhesion molecule (ICAM-1), cytokine (IL-6) and chemokine (IL-8). These TLR2-mediated inflammatory responses in human CAECs share similarities with those induced by TLR4 stimulation with LPS. Importantly, we found that diabetic CAECs have enhanced inflammatory responses to both TLR2 and TLR4 agonists. Therefore, T1D enhances the inflammatory responses to TLR2 and TLR4 stimulation in CAECs. However, this study was performed using cells from a small group of diabetic donors. Large scale studies are needed to further validate these findings. Since chemokines and adhesion molecules play a crucial role in atherogenesis through recruiting inflammatory cells [3] and atherosclerosis decreased in association with reduction of inflammation [32], our results indicate that the pro-inflammatory phenotypic change in CAECs may contribute to the mechanisms underlying the higher risk for atherogenesis in T1D patients. A number of studies demonstrate that T1D has a variety of effect on vascular biology [33]. Recent reports described effects of diabetes on circulating smooth muscle progenitor cell differentiation and vascular smooth muscle cell calcium handling [34,35]. The findings of the present study indicate that T1D may enhance the inflammatory responses of coronary artery to pathogen patterns.

TLR2 recognizes lipoproteins and PGN from gram-positive bacteria, and TLR4 recognizes LPS from gram-negative bacteria [9,36]. It has been reported that higher eukaryotes have other PGN recognition proteins including CD14, Nod1 and Nod2 that induce host responses to bacteria [37]. In addition, CD14 is also involved in cellular responses to LPS [38]. We determined the role of TLR2 and TLR4 in cellular responses to PGN and LPS in coronary endothelial cells. We found that PGN and LPS induced ICAM-1 expression in wild-type cells. However, PGN had no effect on TLR2 KO cells, and LPS had a minimal effect on TLR4-defective cells. These results confirmed that PGN induces the inflammatory responses in coronary vascular endothelial cells through the TLR2 pathway, and the effect of LPS in this cell type is TLR4-dependent.

### The enhanced inflammatory responses in diabetic CAECs are associated with augmented NF-κB activation, but not an alteration of TLR2/4 levels

The main consequence of stimulation of TLR2 and TLR4 is the activation of NF-κB, which mediates the expression of cytokines, chemokines and adhesion molecules [39]. Pro-inflammatory cytokines induced by NF-κB also cause the activation of NF-κB, forming a positive regulatory loop to amplify the inflammatory responses [39,40]. In this study, we found that stimulation of TLR2 or TLR4 induces more pronounced NF-κB phosphorylation and intranuclear translocation in diabetic CAECs. The augmented NF-κB activation should play an important role in the enhancement of inflammatory responses in diabetic CAECs. The mechanisms underlying the enhanced NF-κB activation in diabetic CAECs remain unclear from this study. A change in TLR2/4 distribution, affinity to ligand or signaling to the MyD88 pathway could alter NF-κB activation in diabetic cells.

Interestingly, TLR2 and TLR4 protein levels in diabetic CAECs are not different from those in non-diabetic cells, either in the baseline or after stimulation. The results indicate that the enhancement of TLR2/4-mediated inflammatory responses in human CAECs by T1D does not involve an increase in cellular levels of TLR2 and TLR4 protein. Previous studies found increased levels of TLR2 and TLR4 in circulating monocytes in T1D patients [41]. It appears that the impact of T1D on cellular TLR2 and TLR4 levels is specific to certain cell types. It remains unclear from this study how NF-κB activation is augmented in T1D CAECs. One possible mechanism is that the availability of TLR2/4 is altered. Further studies are needed to examine whether subcellular distribution of TLR2 and TLR4 is altered in diabetic CAECs. Alternatively, T1D may alter the efficiency of post-receptor signaling. TLR2 and TLR4 share the myeloid differentiation primary response gene (MyD)88-mediated pathway to activate NF-κB. In this signaling pathway, MyD88 recruits interleukin-1 receptor-associated kinase (IRAK), leading to the activation of tumor necrosis factor receptor-activated factor (TRAF)-6, and TRAF-6 subsequently activates NF-κB [42,43]. It is possible that the MyD88 pathway becomes more efficient to transduce TLR2 and TLR4 signal in diabetic cells. In this regard, reactive oxygen species (ROS) plays a critical role in activating pro-inflammatory signaling pathways downstream of TLR2 and TLR4 [44,45] and enhances the activity of NF-κB [46]. Elevated generation of ROS in diabetic CAECs in response to TLR2 and TLR4 stimulation may augment NF-κB activation and the resultant expression of inflammatory mediators. Nevertheless, further studies are needed to explain why diabetic CAECs exhibit enhanced TLR2/4 responses in the absence of increased levels of these receptors.

### Insulin alone is insufficient to suppress the hyper-inflammatory responses in diabetic CAECs

There are distinct insulin receptors and post-receptor signaling pathways in CAECs [47,48], and atherosclerotic lesions are worsened in mice lacking endothelial insulin signaling [48]. To determine whether the enhanced inflammatory responses could be corrected by insulin, we stimulated T1D cells with PGN and LPS in the presence of insulin. The normal range of blood insulin has been reported to be 7-24 mU/l, and treatment with 10 U/l of insulin reduces the levels of glycosaminoglycan in cultured endothelial cells [49]. We applied insulin at 10 and 100 U/l to diabetic cells prior to stimulation with a TLR2 or TLR4 agonist. We found that insulin at 10 U/l had no effect on ICAM-1, IL-6 and IL-8 levels following stimulation with either PGN or LPS. Higher concentration of insulin (100 U/l) did not affect LPS-induced production of ICAM-1, IL-6 and IL-8 although it attenuated PGN-induced ICAM-1 and IL-6 production. Therefore, insulin alone in a concentration of 10 U/l could not correct the hyper-inflammatory responses to both TLR2 and TLR4 agonists. A higher concentration (100 U/l) of insulin had no effect on TLR4-mediated inflammatory responses although it reduced ICAM-1 and IL-6 levels following TLR2 stimulation. It has been reported that a large dose of insulin attenuates systemic inflammatory response in endotoxemic mice [50]. It is likely that insulin is potent in suppression of the TLR4-mediated inflammatory response in circulating leukocytes. Since cells are treated with insulin in the absence of glucose, it remains unclear whether a lower concentration of insulin, in the presence of glucose, suppresses the inflammatory response in diabetic CAECs.

## Conclusions

In conclusion, the results of the present study show: 1) stimulation of TLR2 and TLR4 induces greater expression of IL-6, IL-8 and ICAM-1 in T1D CAECs, 2) the enhanced inflammatory responses to TLR2 and TLR4 agonists in diabetic CAECs correlate with augmented NF-κB activation in the absence of an alteration of cellular TLR2 and TLR4 protein levels, and 3) insulin alone is insufficient to suppress the hyper-inflammatory responses to both TLR2 and TLR4 agonists in diabetic CAECs. Since CAECs have an important role in the development of atherosclerosis, an inflammatory disease [51,52], our findings suggest that the pro-inflammatory phenotype of T1D CAECs may be one of the factors contributing to the higher risk for coronary artery atherosclerosis in T1D patients.

## List of abbreviations

CAECs: coronary artery endothelial cells; ICAM-1: intercellular adhesion molecule-1; LPS: lipopolysaccharide; PGN: peptidoglycan; ROS: reactive oxygen species; TLRs: Toll-like receptors; T1D: Type 1 diabetes; TLR2 KO: Toll-like receptor 2 knockout.

## Competing interests

The authors declare that they have no competing interests.

## Authors' contributions

JL is involved in experimental design, acquisition and analysis of data, and drafted the manuscript. CJ and AL participated in the acquisition and analysis of data. JC and DX were involved in drafting the manuscript. DF and XM participated in designing the experiments and revised the manuscript. All authors read and approved the final manuscript.
